# The Middle East and COVID-19: time for collective action

**DOI:** 10.1186/s12992-021-00786-1

**Published:** 2021-11-22

**Authors:** Louise Fawcett

**Affiliations:** Department of Politics and International Relations, St Catherine’s College, University of Oxford, Oxford, OX1 3UJ UK

**Keywords:** Middle East and North Africa, COVID-19, Inequality, Conflict, Regional cooperation

## Abstract

**Revised: Nov 6 2021**

The shortfalls of multilateral and regional organizations in respect of handling the COVID-19 pandemic have been well rehearsed by scholars and policy makers in multiple publications and statements. While the World Health Organization (WHO) and its regional offices have coordinated global responses, regional organizations, like the European Union, Association of Southeast Asian Nations, or African Union, have played complementary roles. However, the response of different regions has varied, revealing multiple deficits in the structures of regional governance. The Middle East and North Africa (MENA) is a region affected by chronic ongoing conflicts and serious inequalities in health and welfare provision, reflected in the absence of concerted responses to the pandemic. Its young population has meant lower comparative mortality rates, but the socio-economic spill-over effects are grave in terms of interrupted education, high unemployment, particularly in respect to vulnerable communities like refugees and migrant workers. With the current situation remaining critical, this paper reviews the impact of COVID-19 on MENA and considers the variable performance of states and institutions to the pandemic, highlighting the shortfalls, but also opportunities for collective action. Drawing on data from the WHO, United Nations (UN), regional organizations, media and secondary sources, it first discusses the wider global-regional context; second, reviews the actions of regional bodies, like the League of Arab States, Gulf Cooperation Council and the cross-regional Organization of Islamic Cooperation; and third, looks at some country-specific situations where both evidence of good practice and the absence of appropriate regional level provision have exposed deep regional divides. It concludes with a call for more collaboration between states and international organizations: better regional coordination is urgently needed to supplement existing multilateral efforts. A collective local response to the COVID-19 pandemic could help transcend regional divides and spur much-needed security cooperation in other areas.

## Background

COVID-19 arrived early to the Middle East and North Africa (MENA) region, with Iran recording the first cases in February 2020. These quickly spread to neighbouring states of the Gulf through business contacts and religious tourism. Soon all MENA countries had reported cases; war-torn Yemen was the last country to record its first case on 9 April 2020. There were prompt responses by most states to control infections, including school, mosque and business closures, lockdowns and strict border controls [1]. Such measures, however, were poorly coordinated and unevenly applied and did not prevent the onset of subsequent waves. By early August 2021 [2] the World Health Organization’s Regional Office for the Eastern Mediterranean (EMRO), incorporating the majority of MENA states, had reported 12.6 m cases and 236 k deaths, though in some countries significant underreporting, whether based on lack of testing, government opacity or incomplete data collection is likely; the overall vaccination rate was then less than 6.0% [3].

The effects of Covid-19 have varied widely across the region. Some states, including Israel and the Gulf states dealt relatively well with early containment and prevention measures followed up by extensive vaccination programmes. By early November 2021 the United Arab Emirates (UAE) and Israel reported 86% and 87% full vaccination rates respectively; with other states lagging far behind, revealing major and continuing discrepancies as well as widespread reported vaccine hesitancy [4]. In contrast to Israel’s figures, the Occupied Palestinian Territories (OPTs) had vaccinated only 25% of the population by early November; Iraq less than 11%. Some states, including Egypt, Iran, Morocco and the UAE, have initiated local vaccine production, others have depended upon imports from China, Europe, Russia, or the US; still others on the COVAX programme. In general, hospitalisation and mortality rates have been lower than other regions, partly due to the very young regional population. With around 60% of the population under the age of 25, MENA is one of the most youthful regions in the world. However, it is also one of the most unequal, and the socio-economic consequences in terms of lost GDP, food insecurity, interruptions to education and unemployment and the impact on vulnerable communities - for example migrant workers and refugees – are particularly concerning [5]. And healthcare systems remain inadequate, even among the wealthiest states; overall the region lags behind its comparable peers.

Alongside other world regions, July–August 2021 saw sharp increases in COVID-19 cases, especially in Libya, Iran, Iraq, and Tunisia, but with numbers also rising in Lebanon and Morocco. Tunisia, a country witnessing political unrest, partly due to popular discontent at government handling of the pandemic, reported record cases and fatalities. A statement in July by Dr. Ahmed Al-Mandhari, WHO Regional Director, described the situation as critical [6]. Statistics since August 2021 reveal a declining case load in most countries and rising vaccination rates, but the pattern of sharp inequalities in provision, alongside high reported caseloads, in war zones like Northern Syria, remains.

The urgency of the situation in MENA is further highlighted by the presence of longstanding conflicts and regional divides which have greatly weakened state capacity and health provision in some cases and reduced the prospects of effective regional cooperation. It is particularly serious in those countries already experiencing the effects of conflict or economic crisis. Both Libya and Iraq are still suffering from the effects of war and fragile infrastructure – in Iraq two major fires swept through critical hospital facilities in April and June 2021 killing at least 200 people. Yemen has been described as the world’s worst humanitarian disaster, with 80% of its population requiring assistance, though the pandemic registers lower on the scale of threats compared to widespread hunger and other disease [7]. In Syria, the results of ongoing war have reduced hospitals and primary care facilities by up to 40%; many workers in the health sector are refugees or among those displaced. Lebanon, amid an ongoing economic and political crisis, in August 2020 saw a massive explosion in the port area of the capital, Beirut, which reduced its hospital capacity by over 30%; Iran is subject to US economic sanctions which limit its ability to access vital equipment and medicines. Across the region, there are millions of internally displaced people and refugees, with camps in Iraq and Syria as well as in neighbouring Jordan, Lebanon, and Turkey placing additional demands on fragile infrastructure. Available data on refugee camps remains limited, but beyond healthcare, scholars have pointed to the additional challenges that the pandemic has posed to protecting the rights of vulnerable refugee populations where host states have restricted movement and access [8]. The gravity and inequality of the current situation cannot be overestimated and is magnified still further by the warnings of the massive potential impact of climate change on the region.

The regional offices of the WHO, the EMRO and EURO, remain principal points of reference in terms of data collection regarding infection, mortality and vaccination rates, but there are several regional and sub-regional bodies, representing the Arab states, the Gulf and Maghreb, as well as a cross-regional Islamic body – Organization of Islamic Cooperation (OIC), which have also played roles in sharing information, providing policy advice and assistance, or in acting as conduit points for international efforts. However, in contrast to other regions – Europe, Africa or Southeast Asia - where collective responses, albeit uneven, have been more robust [9], organizations in the MENA region are weak; hampered by internal divides and ongoing conflicts, and this results in weak policy coordination across multiple issue areas. There are some promising initiatives, particularly in the Gulf, but there has been little sustained cooperation in terms of control measures, procurement and distribution of essential supplies including vaccines. Much of the effort in the MENA region has hitherto resided with individual states, often with support from external partners notably China, Russia, the EU and the US - providing a geopolitical dimension to the region’s responses to the pandemic. China, whose economic links with the region have grown steadily in recent years, has provided extensive technical support and assistance since the early stages of the epidemic. The EU has committed considerable resources to its Southern Mediterranean neighbours, Algeria, Morocco and Tunisia. The Moroccan government, for its part, has signed an agreement with China to start local production of the Sinopharm vaccine [10]. Egypt, with the largest population in the region (100 m), aims to produce both the Chinese (Sinovac) and Russian (Sputnik) vaccines by the end of 2021 to accelerate its hitherto slow vaccination programme. Similarly, Iran has received technical assistance from Russia and Cuba in developing and producing its own vaccines, though roll out so far, has been slow, as discussed below [11]. However, despite such piecemeal achievements and multiple bilateral arrangements, there is no common platform or strategy for regional states to address ongoing problems.

This paper outlines the efforts of regional organizations as well as those of some individual states, highlighting the many challenges as well as possible arenas of cooperation on which to build. There is recent scholarship on the shortfalls of regional cooperation in MENA revealing its poor performance on most indicators against comparable organizations around the world [12]. Persistent conflict, authoritarian regimes, the absence of leadership and external interference have all been offered as explanations for the failure of regionalism. This conclusion is corroborated by recent literature on state and regional responses to the COVID-pandemic from a variety of sources [13]. All commentators agree on the shortfalls of collective action and the need for more regional initiatives to support member states and multilateral institutions in health and other areas: a crisis of this nature offers an important opportunity to put political divisions aside and focus on the benefits collaboration. This paper provides a critical commentary on the region’s response to the COVID-19 challenge. Drawing on data from the WHO, the UN, regional organizations, research institutes, media outlets, and secondary sources, it first discusses the Middle East situation within the wider global-regional context; second, reviews the actions of regional bodies, like the League of Arab States, Gulf Cooperation Council or the OIC; and third, looks at some country specific situations where both evidence of good practice and the absence of appropriate regional level provision have exposed deep divides. It concludes with a call for better coordination among both regional and international actors, including a ‘pan-regional’ effort at cooperation, recalling the initially successful, and wide-ranging multilateral conferences which followed the 1991 Madrid Conference to initiate a Middle East peace process (or the Conference (later Organization) of Security and Cooperation in Europe following Helsinki Accords in 1975 which contributed to the ending of Cold War tensions). In a region where cooperative mechanisms are weak, there is compelling evidence that better coordination is urgently needed to complement existing multilateral efforts and build bridges between states. A collective local response to the COVID-19 pandemic could help to transcend regional divides and thereby spur much-needed security cooperation in other areas, to kick start a new, ‘Middle East security architecture’ [14] – one that would embrace a wide basket of issues from conflict reduction and climate change to all aspects of ‘human security’ including health [15].

## Main text

### Regional-global context

While multilateral organizations like the UN and WHO, alongside states, are seen as the primary security providers in international politics, the role of regional organizations in complementing such efforts is longstanding. The UN family includes six regional economic commissions; its Charter makes explicit provision for action by regional agencies in Chapter VIII [16]. The WHO, in turn, has six regional offices. Despite long-expressed preferences for multilateral action – or the principle of ‘universality’ – the reality has always been subsidiarity, or ‘sub-contracting’ - burden sharing with states and regional bodies [17]. This subsidiarity has always been present but became particularly visible in the post-Cold War period when there were calls for regional actors to take on more significant roles in security provision. Multiple ‘peace operations’ were designed to address a range of new security issues from humanitarian intervention to state reconstruction, particularly in parts of Europe, like the former Yugoslavia, and Africa, as in Liberia and Sierra Leone. There is today huge diversity among regional organizations around the world, and in their relationship with multilateral institutions, but scholars and policymakers acknowledge the importance of an integrated approach in dealing with pressing global security questions including conflict, migration, environmental and health, to name just a few. This is particularly urgent in the Global South [18]. Health issues are present today on the agendas of most regional organizations, and it is notable that some with recent experience of major health crises, including SARS, MERS, Ebola and Zika, have acted to upgrade provision and thus been better prepared, Southeast Asia is one such example [19]. Given the much-anticipated global nature of the COVID-19 pandemic, it might reasonably be expected that most, if not all, regional organizations would have upgraded provision appropriately. However, so far results remain patchy, even among like most advanced regional organizations like the EU, and nowhere more so than MENA, despite its own recent experience with handling the MERS epidemic [20].

### Regional organizations in MENA

Principal regional bodies within the MENA region include the League of Arab States (LAS), the Gulf Cooperation Council (GCC), the currently largely moribund Arab Maghreb Union (AMU), and the cross-regional OIC which includes all MENA states except Israel. Other regional associations and partnership programmes exist linking the region with external partners like the EU, US, Russia and China. Many of these partnerships have been important in supporting pandemic responses, but the focus here is on groupings comprising regional states and on regional states lying outside such groupings. Indeed, one unusual feature of the MENA region, as defined, is that it includes three states which fall entirely outside the relevant (Arab only) regional bodies – Iran, Israel and Turkey. These three countries, which between them have eight Arab neighbours, (more if the Gulf littoral states are included) have individual bilateral relations with some Arab states but are not part of any wider regional framework. Given the region’s hitherto weak record in terms of regional organization, and the absence of any cross-regional platform, it is unsurprising that responses to the pandemic have been uneven and largely reliant on individual states, external partners and multilateral institutions.

*The League of Arab States* (LAS) is the oldest regional organization, founded in 1945 after the Second World War, comprising the 22 mainly Arab-speaking countries, thereby covering all states of the region except Iran, Israel and Turkey. The latters’ very exclusion from the regional body is one evident limitation but the League’s scope of action in recent decades has been hampered by inter-Arab divides since the two Gulf Wars, the MEPP, the Iraq War and the Arab uprisings. Syria’s membership remains suspended due to disagreement over the status of President Assad’s regime. From its establishment, the LAS has served more as inter-governmental forum rather than a mechanism for promoting integration, but it has coordinated policy on certain issues, notably the Palestine question, and supported some post-conflict operations. Its founding Charter includes a commitment to health cooperation, but given its ongoing weaknesses, the Covid-19 pandemic did not meet with a rapid, or robust response [21]. Indeed, the League’s scheduled 2020 summit, rather than providing an opportunity for health cooperation, was cancelled. Nonetheless, the LAS has served as a forum for discussion of COVID-19 questions, while issuing several declaratory statements. It hosted an early conference between Arab states and China to share information and discuss collaboration; another ongoing UNDP-sponsored initiative has brought LAS states together with Japan in a series of high-level meetings to address the UN’s Sustainable Development Goals for the Arab world in the light of the COVID pandemic [22]. Similarly, the UN Office of Disaster Risk Reduction (UNDRR) has co-organized events with Arab countries to share COVID lessons and explore mitigation measures within a wider discussion of risk mitigation measures [23].

Despite use of the LAS forum to highlight ongoing issues, however, the tendency has been for Arab states to design their own policies, often with the technical support and assistance of outside powers, and here both China and Russia have been important players, promoting their regional interests in what scholars have called ‘vaccine diplomacy’ [24]. Some Arab countries have provided support to their neighbours and states further afield, with the Gulf states, notably the UAE, particularly active in this regard. Egypt has signalled that once its own vaccine production is underway it will start regional distribution. Still, there is an evident absence of concerted policy or leadership leaving the Arab world ‘between a rock and hard place’ in the words of one commentator [25].

*The Gulf Cooperation Council* (GCC), a sub-regional group comprising six Gulf littoral states: Bahrain, the Kingdom of Saudi Arabia (KSA), Kuwait, Oman, Qatar and the UAE, provides a useful point of contrast with the larger Arab body. The GCC, with an overall population of $58.5 m (and GDP of $5.5 bn) has been the most effective regional organization to date, despite inequalities and ongoing divides between two members, Saudi Arabia and Qatar. Formed in 1981, partly to secure the Gulf states against their powerful neighbours, Iraq and Iran, following Britain’s withdrawal from the Gulf region, it includes mostly high-income states, many with comparatively well-developed health and welfare systems. Common regime type, and economic and social linkages have helped to build functional cooperation and business networks, and the GCC has been regarded as the region’s most successful regional organization, until a divisive split occurred between Qatar and KSA in 2017. One important element from a health perspective is the region’s prior experience with MERS, a virus transmitted to humans through infected camels first reported in 2012, with KSA being the most affected state. Research showed variable public understanding of MERS, but preventive regional measures were widely adopted to deal with the outbreak – handwashing and mask-wearing for example - and these were undoubtedly a factor in better regional preparedness for COVID-19 [26]. In addition, both KSA and Qatar have advanced and well-funded medical research and technology facilities, with both King Abdulla University of Science and Technology and the Qatar National Research Foundation being major players in the field. Both have supported rapid response COVID-19 programmes. Another factor in regional preparedness was the GCC’s experience with managing large scale tourism and migration. Not only does the Gulf, particularly the UAE, attract large numbers of tourists – it also receives millions of religious pilgrims annually in the Saudi case. Though tourism and the annual hajj were both curtailed during the worst periods of the pandemic, advanced monitoring systems using digital technology were already in place to regulate the movement of visitors and citizens which could be repurposed to control movement during critical periods [27].

Against the above backdrop, there were early responses from GCC countries to successfully ‘flatten the curve’. Promising initiatives at the start of the COVID-19 crisis included the setting up of a Gulf crisis room to coordinate responses and a network to protect food supplies – a critical area given the region’s dependence on external food supply chains [28]. And, as the economic impact of the COVID epidemic became evident, the wealthier Gulf states, including KSA, Qatar and UAE, introduced billion-dollar stimulus packages to help boost economies facing the heavy cost of lockdowns and falling oil prices. Individual states also made extensive use of innovative technology, with Qatar introducing a nation-wide coronavirus tracing app: *Ehteraz* and the UAE rolling out a mass drive-through testing programme [29]. Kuwait’s new healthcare app for women, *Nabta*, also provides users with updated information on COVID-related health issues.

Many of the above measures lie more at the level of individual state policy and do not necessarily provide evidence of enhanced regional, or cross-border cooperation, but given the region’s interdependence, the potential spill-over benefits are considerable. And, there have been other positive indicators, including the lifting of the group’s three-year blockade against fellow member Qatar in early 2021, though for reasons not directly related to the pandemic, freeing the way for bloc-wide cooperation. GCC members have supported negotiations for a ceasefire in Yemen to allow the country better access to humanitarian assistance and respite from its devastating 7-year civil war. There has been increased engagement between Gulf states and Iran, primarily the UAE, which provided Iran early pandemic support, but also, and more tentatively, Saudi Arabia. Relations between these two countries were ruptured in 2016 amid serious differences over competing regional interests in Yemen, Iraq, Lebanon and Syria. Any move to mitigate the Saudi-Iran rivalry will have a positive regional spill-overs and help to support the development of a much-needed regional security system.

The above are piecemeal measures, and, as with handling of the COVID-pandemic, any fully coordinated response has not materialised, despite the potential benefits to all parties. In addition, as noted, pandemic measures have negatively impacted on other areas, like food supplies and labour mobility. Indeed a feature of the pandemic has been to expose the vulnerability of certain sectors of the Gulf population, particularly expatriate workers, on whom states depend for vital services, but who often live in more crowded and less sanitary conditions with unequal access to health provision [30]. The absence of any region-wide mechanism to manage migration and labour conditions for such informal workers (many of whom are women) was a source of concern long before COVID struck, but the pandemic has brought their situation more sharply into focus, with evidence also suggesting that mortality rates among migrant workers have been considerably higher than among local populations. If the GCC, therefore, provides evidence of good practice and the potential benefits of pooling information, technology and resources, the initiative, as with the LAS, still often lies at the level of individual states with significant disparities between them. And critics have also highlighted some negative impacts of the ‘securitization’ of health policies, including the widespread use of health apps, which have increased the power and scrutiny of centralised authoritarian regimes and restricted individual rights and freedoms.

The *Arab Maghreb Union* (AMU), another Arab-only subregional organization, in contrast to the GCC, is remarkable for its failure to design any collaborative initiatives to the pandemic. Founded in 1989, and comprising the five Arab countries of Northwest Africa (Algeria, Libya, Mauritania, Morocco, Tunisia), the AMU displayed early potential in repairing regional divides, building on economic interdependencies and improving relations with European partners. Divisions between key members, notably Algeria and Morocco, over the longstanding Western Sahara dispute, soon resurfaced, however, obstructing progress and keeping leaders away from the summit table. The AMU has existed mostly in name only - as a point of reference for possible coordination but not as an example of it. The World Economic Forum report in 2017 described it as one of the world’s worst performing trading blocs, pointing to multiple lost opportunities [31]. The COVID-19 pandemic represents yet another missed opportunity for Maghreb cooperation. The low-to-middle incomes of Algeria, Morocco and Tunisia have all implemented far-reaching measures to contain the virus which has negatively affected their economies, and in the case of Tunisia, contributed to sustained political unrest, but despite the gravity of the situation faced by the region, its members have been unable to put aside political differences to support a common approach. The region experienced a high caseload in July/August 2021 and, despite a subsequent decline, Libya, and its (non-AMU) neighbour Egypt, continued to experience high rates of infection, while vaccination rates are uneven. Reuter’s early November data showed that Algeria and Libya had only fully vaccinated 13 and 15% of their respective populations in comparison with considerably higher rates in Morocca (65%) and Tunisia (40%) [32].

If the *Maghreb* – representing Western MENA - is currently a region without effective regionalism, the same is true of the Eastern MENA region, or *Mashriq*, the historically and geographically interconnected states of the Eastern Mediterranean, or the Levant – Lebanon, Israel, the OPTs and Syria. Yet despite their many interdependencies, the Levant states lack any relevant grouping or collaborative body. This reflects historic divides between Israel and Arab states, but also between Mashriq states themselves. However, since the bilateral agreements whereby first Egypt (1979), then Jordan (1994), signed peace treaties with Israel, some barriers to cooperation have been removed. (In 2020, the US-brokered Abraham Accords, also saw the UAE and Bahrain enter agreements with Israel.) Israel’s world-leading efforts to combat and contain COVID-19 have been noted, though its early measures did not prevent subsequent waves, and, like other, mainly Western countries, it has been criticised for vaccine nationalism and ‘hoarding’. The conditions in the OPTs differ quite starkly, however, particularly the Gaza Strip with its overcrowded living conditions and limited access to medical supplies and facilities. The OPTs have received support from international bodies like the WHO and the OIC (see below). The situation has deteriorated since the outbreak of new hostilities between Gaza and Israel in May 2021 heightened the disparity of provision and Gaza’s fragile health infrastructure, leading to calls for more robust international and regional action including a rapid roll-out of vaccines to the OPTs. Indeed, the high levels of interdependence between Israel and the OPTs unite them as an ‘epidemiological unit’, making particularly urgent the need for cooperation, whether in respect of medical supplies, data sharing and support for more resilient medical structures [33]. However, so far the COVID pandemic represents another lost opportunity for more creative diplomacy in the longstanding Israeli-Palestinian conflict [34].

Among other Mashriq countries, Jordan’s response to the virus has also been robust, though giving rise to criticism of its aggressive policing of lockdowns and borders; Syria remains a country at war with still limited data available on the impact of the pandemic. In October 2021, however, Medecins Sans Frontiers (MSF) reported on the severity of a new wave which hit Northern Syria [35].

Two other regional states, Turkey and Iran, remain outside any existing MENA grouping (though not the cross-regional OIC discussed below). Like Israel, both have relations with individual Arab states but have also drawn on external support and national self-reliance when it comes to pandemic responses. Though these two countries have experienced the highest caseloads in MENA: 8.1 m and 5.9 m respectively, [36] Turkish fatalities to date (71,000) have been considerably lower than those of Iran (127,000) for reasons that relate to its early lockdown policy and more advanced healthcare provision, though some have questioned the reliability of reported figures [37] Turkey had fully vaccinated over 60% its population by early November. The situation in Iran is particularly complex given the early onset and gravity of the pandemic, its relative regional and global isolation amid ongoing conflicts with Arab states, Israel and the West and the punitive effects of US sanctions. Though Iran’s health sector is comparatively well-developed, as is its research and technology base, its economy is particularly vulnerable due to the extent of the pandemic, the effects of declining oil prices and international sanctions which have limited its access to currency and vital supplies. While adopting a variety of lockdown and preventive measures, perhaps delayed because of the March Nowruz (New Year) celebrations, Iran remains the worst-hit MENA country. It has depended hitherto on vaccine supplies from China, Russia and India, as well as the COVAX programme, but initial vaccination rates were low – around 5% in July 2021 [38]. The locally produced, *COVIran Barakat* became available in June 2021, with Supreme Leader Ayatollah Khamenei publicly receiving an early dose [39]. The expectations were to start producing 3 m vaccines monthly, to be increased to 11 pcm by the end of the year, making it potentially the largest regional vaccine producer and exporter to other states. But Iran’s vaccine roll-out has encountered obstacles, leading to further offers of external assistance, including a significant aid package from Japan, and the reversal of an earlier decision to refuse vaccine imports from the US and UK. Figures indicated that by early November, Iran had come close to vaccinating 50% of its population.

Aside from its own control and prevention measures, any easing of regional tensions would significantly improve Iran’s situation, as would a successful conclusion to continuing talks with the US over the terms of a renewed JCPOA and with it the lifting of international sanctions. Iran has signalled it is willing to engage in regional dialogue, but as 2021 draws to a close, the extent of that commitment, by Iran and other regional and international actors, remains to be tested [40].

*The Organization of Islamic Cooperation (OIC),* finally, deserves mention as a body that crosses different world regions, including MENA, providing an umbrella grouping for Islamic-majority states around the world. A loose intergovernmental body, with a general commitment to economic development and well-being, its charter seeks to develop science and technology and encourage research and cooperation among member states [41]. In this regard the OIC has been an active player in organizing events and workshops, disseminating information and providing support to Islamic states in need. A comprehensive report, issued in May 2020, detailed some of these measures, including assistance to conflict torn and vulnerable states and territories like Palestine and Yemen, creating a platform to encourage information-sharing and good practices, and the launching of a media awareness platform [42]. A follow up report in October 2021, highlighted the impact of the crisis in the critical area of food security among member states [43].

Like the LAS, though more effectively, the OIC has hosted online events, including a meeting with rectors of OIC universities to discuss collective strategies in the fight against COVID-19; another to combat fake news – not insignificant among member states where misinformation and vaccine hesitancy is reportedly high. Via its Islamic Solidarity Fund, it has offered tranches of assistance to OIC members; mainly Least Developed Countries or states with urgent humanitarian needs. Finally, it has regularly endorsed and supported individual state policy, for example in the testing and production of local vaccines, or in adopting COVID-compliant measures in respect of religious activity and festivals.

All the above instances of cooperation, both within and without regional and cross-regional organizations in MENA, offer a snapshot of some of the ongoing efforts to address the COVID-19 pandemic. While these are subject to change and review, they evidently fall short of an effective or collective response and need to be considered alongside other state-led and multilateral efforts. There has been no effective coordination of border controls, quarantine measures or procurement and distribution of medical supplies, including vaccines. Given the shortfalls of regional level action, MENA responses have depended heavily on the efforts of individual states and their overseas allies. The most vulnerable states and communities have depended on the COVAX programme and international assistance from a variety of sources. Of course, other regions, including Europe, have been heavily criticised for their slow and fragmented responses to the pandemic. But MENA stands out as a region where cooperative mechanisms are weak and the demand for collective action high, particularly given the prevailing high levels of inequality and conflict. It has performed less well than peers. Here a contrast with a region like Southeast Asia is instructive. The Association of South East Asian Nations (ASEAN) has a comprehensive and updated web page which effectively track the region’s cases and responses [44]. Another contrast is provided by the far less well resourced, African Union, where the region’s ministers of health have endorsed a coordinated strategy in the fight against COVID [45].

## Conclusion

The COVID-19 pandemic has revealed the centrality of public health to global security, broadly conceived. As WHO director, Tedros Adhanom Ghebreyesus, aptly puts it: ‘No one is safe, until we are all safe.’ However, the refrain that security is ‘indivisible’, has not been sufficient spur to collective action, even over a global crisis of such gravity. The world now faces still graver issues in relation to the threat of climate change – one which the MENA region, again, is currently poorly equipped to address. Indeed COVID-19 has exposed the multiple shortfalls in the structures of multilateral governance and demanded new innovative responses from all states [46]. While the emphasis must be on global provision and equitable distribution, one pathway to achieving this goal is via the regions of the world, as the structures of the UN and WHO recognise. Implementing effective health measures in a global pandemic requires cross-border cooperation, which in turn depends on effective regional institutions. This is as relevant to COVID-19 as it is to other challenges including conflict resolution and climate change. This paper has argued for more integrated responses at the sub-regional and regional level to complement global and state efforts.

The pandemic has presented huge challenges to structures of global governance, but also opportunities for learning and preparing for future crises. Here past lessons may be important. In MENA the situation may be likened to that of the 1990s, when, following the Madrid Peace Conference of 1991, an ambitious multilateral peace process was initiated to accompany the ongoing negotiations between Israel and Arab countries. This multilateral track involved multiple schemes involving Israel, Arab states and international actors to support the peace process, not only to normalise Arab relations with Israel and support the development of a Palestinian state, but also through different working groups on refugees, water, the environment and arms control and regional security: in short, an ambitious effort to help cement a more stable regional order [47]. The multilateral process ultimately faltered, but it is precisely these kinds of iterative and broad reaching confidence building steps that are currently required to regenerate region-wide cooperation [48].

Since 2000, the region has faced a growing spiral of conflict and insecurity with new wars and fresh divides complicating the landscape. Alongside the Israel-Palestine conflict, new issues crowd the regional agenda, from the crisis in Yemen, to conflict in Syria, the GCC split, and the Iran-Saudi rivalry alongside the continuing impasse over Iran’s nuclear programme. The critical situation in Afghanistan, whose health infrastructure has been debilitated by decades of war, poses additional security challenges to MENA states [49]. The Coronavirus pandemic has intervened at a moment when the region had reached a low point in terms of insecurity and regional fragmentation. Political divides within the region, and geopolitical divides without have all obstructed the possibilities of cooperation. Yet global health threats, like environmental threats ‘know no political borders’, and demand effective collaboration from states. Sub-regions, like the Gulf, Levant and North Africa all offer opportunities for cross-border cooperation – opportunities still not taken in most cases – but there is also a need for a wider regional framework to engage all MENA actors. Now is the time to support a renewed multilateral effort, one drawing in major regional and external powers and international organizations, to address multiple problems. It could provide the spark to cooperation that the region needs so badly, by initiating a series confidence-building measures to advance the limited progress already achieved.

This paper has offered an overview of regional developments, largely from the perspective of early August 2021 when much of the region was experiencing the worst effects of the COVID-19 pandemic. Since that date, there has been a slowing-down of cases and higher availability and uptake of vaccines, but the region remains vulnerable to new waves, particularly in conflict areas. Its purpose has been to expose the limits and opportunities for regional collaboration in MENA over the handling of COVID-19. These point to great inequality in provision, variable responses and vaccine roll-out amid an ongoing pandemic and grave regional conflicts involving mass displacement of vulnerable people whose status prior to the pandemic was already precarious. Improved regional cooperation at all levels could secure better cross-border regulation, data sharing and equitable distribution of medical supplies, including vaccines. If the tools for regional cooperation are currently weak, evidence suggests there is considerable capacity for a more robust regional effort to complement ongoing efforts by states and multilateral institutions notably the UN and WHO. Regional states, led by those with the strongest economies – like Israel and the Gulf states - could help to initiate cooperative measures starting from the principle of the lowest common denominator – the right of each MENA citizen to basic health provision and what in UN terms is called ‘human security’. Health cooperation, in turn, could feed into confidence building measures in other pressing areas like mitigating climate change. The time for such cooperation is now.

## Data Availability

There are limitations to the data presented here, based on lack of testing and monitoring facilities in some countries as well as government opacity or incomplete data collection. Data from different sources can also vary.

## Abbreviations

AMU: Arab Maghreb Union
GCC: Gulf Cooperation Council
EMRO: Eastern Mediterranean Regional Office (WHO)
EURO: European Regional Office (WHO)
KSA: Kingdom of Saudi Arabia
LAS: League of Arab States
MENA: Middle East and North Africa
OIC: Organization of Islamic Cooperation
OPT: Occupied Palestinian Territories
POMEPS: Project on Middle East Political Science
UAE: United Arab Emirates
UNDP: United Nations Development Programme
UNDRR: United Nations Office of Disaster Risk Reduction
WHO: World Health Organization

## References

[1] UNDP (2020). Arab states step up response to Coronavirus.

[2] Research for this paper focused on the period July/August 2021. Selective updates have been included until early November 2021.

[3] The EMRO Is one of the six WHO regional offices. It covers 21 MENA states including the Palestinian territories, as well as Pakistan and Afghanistan, with an overall population of 679m: http://www.emro.who.int/index.html . EMRO excludes Turkey and Israel which are included in the WHO’s European Regional Office (EURO): https://www.euro.who.int/en/home;

[4] Reuters. COVID-19 Tracker, Asia and the Middle East: https://graphics.reuters.com/world-coronavirus-tracker-and-maps/regions/asia-and-the-middle-east/; EMRO. Statement on COVID-19 in the Eastern Mediterranean Region August 2021: http://www.emro.who.int/media/news/statement-on-covid-19-in-the-eastern-mediterranean-region-2-august-2021.html Arab Barometer. Vaccine Hesitancy in MENA. July 2021: https://www.arabbarometer.org/2021/07/vaccine-hesitancy-in-mena/

[5] OECD (2020). COVID-19 response in MENA countries.

[6] EMRO (2021). COVID-19 situation critical in Eastern Mediterranean region.

[7] Alsabri M (2021). Conflict and COVID-19 in Yemen, Global Health.

[8] Barnes J, Makinda M (2021). A threat to cosmopolitan duties? How COVID-19 has been used as a tool to undermine refugee rights, international affairs, 97/6.

[9] Alcano R, Tocci N (2021). Navigating a COVID World, International Spectator.

[10] Reuters (2021). Morocco’s Sothema to produce China’s Sinopharm vaccine.

[11] Times T (2021). Tehran-Havana strategic cooperation on coronavirus vaccine production.

[12] Del Sarto R, and Soler y Lecha E. The Mirage of Regionalism in MENA. MENARA PROJECT 2018: http://diana-n.iue.it:8080/handle/1814/65449 Fawcett L. Regionalism and Alliances in the Middle East. International Relations of the Middle East Oxford 2019. 10.1093/hepl/9780198708742.003.0010 Valbjørn M. The Middle East and North Africa. Oxford Handbook of Comparative Regionalism. Oxford 2016: 10.1093/oxfordhb/9780199682300.013.13.

[13] Eg, POMEPS, The Covid-19 Pandemic and the Middle East and North Africa, No. 39, April 2020: https://pomeps.org/wp-content/uploads/2020/04/POMEPS_Studies_39_Web.pdf Alden C, and Dunst C, The Middle East and The Arab League and Covid 19: https://www.lse.ac.uk/international-relations/centres-and-units/global-south-unit/COVID-19-regional-responses/Middle-East-and-COVID-19; Karamouzian M, Madani M. COVID-19 response in Middle East and North Africa. Lancet. May 2020: https://www.thelancet.com/journals/langlo/article/PIIS2214-109X(20)30233-3/fulltext; Altunışık M, Pandemic Regionalism, or Not? The MENA Region in the Shadow of COVID-19, International Spectator 2021; 10.1080/03932729.2021.1903730

[14] Jones P (2020). Time to Establish a Middle East Security System’. Cairo Review.

[15] See UN trust fund on Human Security https://www.un.org/humansecurity/what-is-human-security/

[16] UN Charter: https://www.un.org/securitycouncil/content/regional-arrangements-chapter-viii-un-charter

[17] Weiss T, Beyond UN (1998). Sub-contracting, Palgrave.

[18] Amaya AB, De Lombaerde P (2021). Regional cooperation is essential to combatting health emergencies in the global south. Glob Health.

[19] Anthony M (2021). COVID-19 in Southeast Asia: pandemic preparedness matters, Brookings.

[20] WHO, Middle East Respiratory Syndrome, https://www.who.int/health-topics/middle-east-respiratory-syndrome-coronavirus-mers#tab=tab_1

[21] Alden C, and Dunst C, The Middle East and The Arab League and Covid 19: https://www.lse.ac.uk/international-relations/centres-and-units/global-south-unit/COVID-19-regional-responses/Middle-East-and-COVID-19

[22] UNDP (2020). LAS-Japan-UNDP Roundtable discusses support for SDGs.

[23] UNDRR 2020, https://www.undrr.org/event/fifth-arab-regional-platform-disaster-risk-reduction

[24] https://www.mei.edu/publications/vaccine-diplomacy-mena-region

[25] Najib D (2021). Covid-19 and the Arab world: between a rock and a hard place.

[26] Public response to MERS in 6 countries, Jnl Infection and Pub Health. 2017: https://www.sciencedirect.com/science/article/pii/S1876034117300278 Alagisi A, et al. Preparedness and response to COVID-19 in Saudi Arabia: building on MERS experience, Jnl Infection and Pub Health, 2020: https://www.sciencedirect.com/science/article/pii/S1876034120304664 10.1016/j.jiph.2020.04.016 PMC7211706 32451260

[27] IISS (2020). Exploring gulf responses to COVID-19.

[28] Brookings 2020; https://www.brookings.edu/opinions/how-gulf-states-can-lead-the-global-covid-19-response/; E Woertz. Wither the self-sufficiency illusion? Food security in the Arab Gulf States and the impact of COVID-19. Food Security, 2020: 10.1007/s12571-020-01081-4, 4, 757, 760. 10.1007/s12571-020-01081-4 PMC7340854 32837639

[29] Al-Hosani F, et al. Review of COVID-19 testing in the UAE. Front Public Health. 2021; https://www.frontiersin.org/articles/10.3389/fpubh.2021.661134/full. 10.3389/fpubh.2021.661134 PMC8149747 34055725

[30] Brookings’s blog (2020). Pandemic highlights the vulnerability of migrant workers in the Middle East.

[31] World Economic Forum (2017). The Maghreb Union is one of the World’s Worst Trading Blocs. World Economic Forum.

[32] Reuters. COVID-19 Tracker, Asia and the Middle East: https://graphics.reuters.com/world-coronavirus-tracker-and-maps/regions/asia-and-the-middle-east/

[33] Negev M (2021). Regional lessons from the COVID-19 in the Middle East. Science Total Environment.

[34] L Lehrs, Conflict and Cooperation in the Age of COVID-19 – the Israeli-Palestinian case. International Affairs 97/6 2021: 10.1093/ia/iiab143.

[35] MSF (2021). Worst wave yet of COVID-19 in Northern Syria overwhelms health system.

[36] All figures from 5 November: John Hopkins Coronavirus Resource Center: https://coronavirus.jhu.edu/map.html

[37] Tank P (2020). Turkey and COVID-19. PRIO MEP policy brief.

[38] https://reliefweb.int/report/iran-islamic-republic/islamic-republic-iran-receives-second-delivery-covid-19-vaccines

[39] Motamedi M (2021). Iran’s supreme leader receives local COVID vaccine. Al Jazeera.

[40] Iran is serious about regional dialogue. Tehran Times. April 2021: https://www.tehrantimes.com/news/460258/Iran-is-serious-about-regional-dialogue

[41] OIC Charter: https://www.oic-oci.org/upload/documents/charter/en/oic_charter_2018_en.pdf

[42] OIC (2020). Efforts of the OIC and its organs in serving Islamic causes and addressing the effects of the Novel Coronavirus pandemic (COVID-19).

[43] OIC (2021). Covid Pandemic compounded food crisis in member states.

[44] COVID-19 Situational Report in the ASEAN region, 11 2021: https://asean.org/wp-content/uploads/2021/10/COVID-19_Situational-Report_ASEAN-BioDiaspora-Regional-Virtual-Center_11Oct2021-1.pdf

[45] Union A (2021). African ministers of health endorse adapted strategy to fight COVID-19.

[46] Meyer T (2021). Towards a New Multilateralism, Routledge.

[47] E Solingen, The Multilateral Arab-Israeli Negotiations. Journal of Peace Research 37/2: 10.1177/0022343300037002004

[48] Vakil S, Quilliam N (2021). Steps to enable a Middle East regional security process.

[49] Essar MY, Hasan MM, Islam Z, Riaz MMA, Aborode AT, Ahmad S (2021). COVID and multiple crises in Afghanistan: an urgent battle. Confl Heal.

